# The influence of region of interest width in fetal 2D-speckle tracking echocardiography late in pregnancy

**DOI:** 10.1007/s10554-021-02455-1

**Published:** 2021-11-03

**Authors:** Tom Roar Omdal, Umael Khan, Cathrine Ebbing, Jörg Kessler, Henriette Odland Karlsen, Elisabeth Leirgul, Knut Matre, Gottfried Greve

**Affiliations:** 1 Department of Heart Disease, Haukeland University Hospital, 5021 Bergen, Norway; 2 Department of Clinical Science, University of Bergen, Bergen, Norway; 3 Department of Obstetrics and Gynecology, Haukeland University Hospital, Bergen, Norway

**Keywords:** Echocardiography, Speckle tracking, Region of interest, Strain, Fetal

## Abstract

Speckle tracking echocardiography is a promising method for assessment of myocardial function in fetal and neonatal hearts, but further studies are necessary to validate and optimize the settings for use in fetal cardiology. Previous studies have shown that the definition of the region of interest (ROI) affects strain values in adults. The aim of this study was to investigate how different widths of ROI influences measurements of four-chamber longitudinal systolic strain in fetuses late in pregnancy. Thirty-one singleton, healthy fetuses born to healthy mothers underwent an echocardiographic examination during gestational week 37. Speckle tracking was performed with two different settings for ROI width; the narrowest and second most narrow, provided both widths were assessed as suitable for the myocardial wall thickness of the fetus. We found an inverse correlation between the ROI width and the strain values. Four-chamber longitudinal strain changed from − 20.7 ± 3.6% to − 18.0 ± 4.4% (p < 0.001) with increasing ROI width. Further, strain decreased from the endocardium to the epicardium with multilayer measurements. Different widths of ROI influenced the strain measurements significantly in the fetal heart, comparable to what has been reported in adults. A standardization of the ROI setting could improve the interpretation, and reduce variability in fetal strain measurements.

## Introduction

Echocardiography is the primary diagnostic tool for evaluating myocardial function. As a supplement to traditional echocardiographic measures of ventricular function such as ejection fraction and fractional shortening, assessment of myocardial strain through speckle tracking echocardiography (STE) is established in adults [1, 2]. It is also a more reproducible method for measuring ventricular function than ejection fraction [3]. STE is also gradually being more used in younger patients [4] and in fetuses [5–9]. Correct clinical assessment of fetal cardiac function is essential for appropriate diagnostics and follow-up in fetuses with impaired cardiac function, in order to plan delivery and treatment.

However, optimal application of this technique requires insight into how image acquisition and processing parameters affect the strain measurements in the fetus and newborn. Previous studies have shown the impact of heart rate [10], transmitting frequency [11], smoothing settings [12], drift compensation [13], angle of insonation [14], frame rate [14] and intervendor variability [15] on strain measurements. However, there is still a gap in knowledge regarding technical challenges associated with the perinatal age group and further research in this age group is needed [16]. Implementing the method of STE in fetuses and neonates requires further investigation to validate and optimize the imaging acquisition and processing parameters.

STE is based on the tracking of the movement of the interference patterns arising from scattered ultrasound waves. It has been shown to detect reduced myocardial systolic function at an earlier stage than traditional measurements of ejection fraction using 2D-echocardiography [17].

When conducting STE, the region of interest (ROI) must be defined. The ROI is the area in the image where speckle tracking occurs, and usually involves some form of delineation of the myocardium. Previous studies of adults have shown that the ROI width affects strain values and therefore is a source of variation for strain measurements [18–20]. The effect of ROI width is further nuanced when layer specific strain is taken into account, with ROI width having an increasing impact on strain as one moves from the endocardial to the epicardial layer. It is established that strain decreases from the endocardial to the epicardial layer in larger hearts [21–23]. However, this has not been investigated in fetal hearts. The aim of this study was to assess the effect of different settings of ROI width on strain measurements in fetal hearts.

## Methods

We invited 39 healthy women with low-risk pregnancies to participate in this observational study. Sample size calculations were conducted, α = 0.05 and β = 0.8, for paired variables. The minimum effect size was set at mean difference of 2%. A pilot study of five fetuses was performed in order to gauge the expected variability for sample size calculations. A dropout buffer of 20% was included. All pregnancies were uncomplicated and all had a normal routine anatomy scan at gestational age 17–20. The fetal echocardiographic examinations were performed during gestational week 37, during the period from June 2017 to June 2019. The study was approved by the Regional Committee for Medical and Health Research ethics (No. 2015/1918), and all participants gave written consent to participate.

Experienced fetal medicine specialists (JK and CE) performed the image acquisitions. The mothers were in a supine position with a pillow under the knees. The fetuses were examined using a Vivid E9 scanner with a 4 MHz cardiac sector transducer (M4S, GE Vingmed ultrasound, Horten, Norway). The probe was chosen to enable sufficient frame rate for the deformation analysis. For the strain analyses, focused B-mode images of the fetal heart in four-chamber (4Ch) view were acquired. Cine loop images of 3 s duration were digitally stored and later analysed using EchoPAC v203 (GE Vingmed Ultrasound, Horten, Norway).

An experienced cardiologist (TRO) analysed all echocardiograms. The beginning of the cardiac cycle was defined by the closure of the mitral valve, representing end-diastole as described in the consensus document by Voigt et al. [24] and by Enzensberger et al. [25]. The end of one cardiac cycle was defined by the frame just before the next mitral valve closure. The ROI was then defined at end-diastole by tracing the endocardial border from the hinge point of the anterior mitral leaflet along the interventricular septum to the apex, continuing along the anterior free wall to the hinge point of the posterior mitral leaflet. The most narrow ROI was chosen as default width. If a segment was defined untrackable by the software, a visual assessment of the segment was performed. When suboptimal tracing was considered due to suboptimal endocardial delineation, the actual segment was repositioned manually, correcting the endocardial markers. If repositioning was not possible, the case was excluded from the analysis. The software calculates strain values in six segments; basoseptal, midseptal, apicoseptal, apicolateral, midlateral, and basolateral, as well as an average 4-chamber longitudinal strain (4ChLS) across these segments. Further, the software would define three myocardial layers; endocardial, midwall, and epicardial. Temporal and spatial smoothing as well as drift compensation were kept at default settings.

**Fig. 1:**
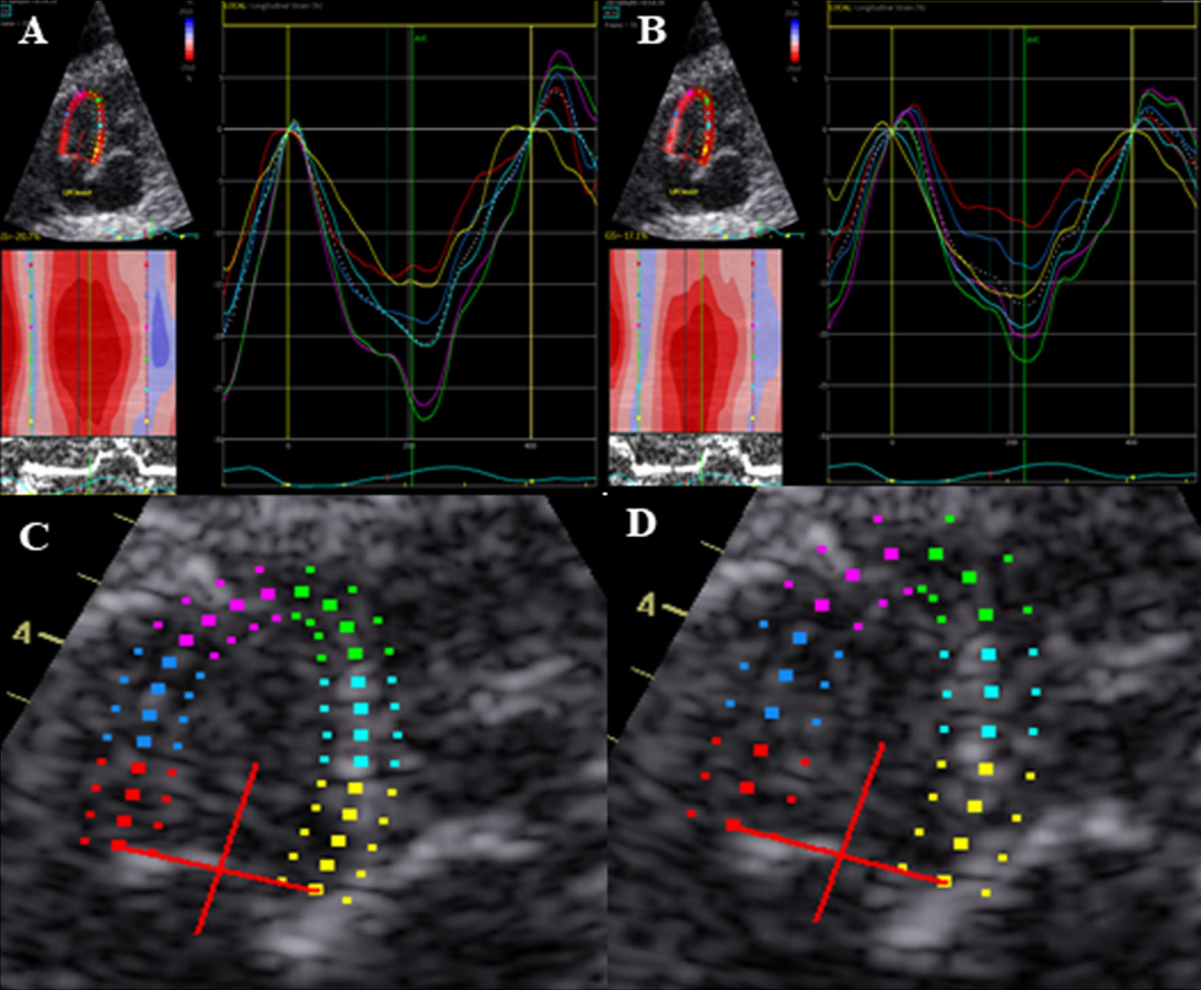
Comparison of the two region of interest widths i.e., narrow (**A** and **C**) and wider (**B** and **D**). When adjusting ROI one step wider, the inner line of the endocardial border remains unchanged while the outer line exceeds the originally defined epicardial border

In the Q-analysis program in EchoPAC, the operator has the opportunity to choose between six defined ROI widths. The numeric dimensions of the six widths are not indicated from the vendor. We considered the two narrowest widths to be most suitable for the wall thickness of the fetal heart. According to the recommendations by Voigt et al. [24] as well as the vendor software instructions, the ROI should not include the pericardium as this could result in underestimated myocardial strain. Hence, we assessed all images at these two settings for ROI width and compared strain values at both widths. The same endocardial tracing was used to assess both ROI widths, ensuring that changes in strain were due to changes in ROI rather than tracing errors. Figure 1 shows an example of ROI for narrow (A, C) and wider (B, D) tracing and the corresponding strain curves for the six segments.

The ROI width was adjusted twice, resulting in three values of strain. After tracing the endocardium, the narrowest ROI width was chosen first. Thereafter, the width was adjusted one step wider. Finally, the width was adjusted back to the narrower setting, resulting in the strain values; narrow, wider, and narrow^*^. The latter adjustment was done as a measure of reproducibility.

### Statistical analysis

A two-way repeated measure ANOVA with Bonferroni post-hoc multiple contrast tests was performed to assess the effect of ROI width and wall layer on 4ChLS values, as well as the interaction between these two effects. The assumption of normal distribution was assessed using the Shapiro-Wilk test of studentized residuals. Violations of normality were addressed by using pairwise Wilcoxon signed rank test. In case of violation of the assumption of sphericity, assessed by Mauchly’s test of sphericity, the Greenhouse-Geisser adjustment was applied. Outliers were defined as studentized residuals greater that ± 3. Reproducibility was assessed using two-way mixed absolute agreement intraclass correlation coefficients (ICC). We selected 25 random recordings for the assessment of intra- (TRO) and interobserver (TRO and GG) variability. The analyses were carried out with the SPSS statistical package version 25 (SPSS Inc, IBM Corp., Armonk, NY, USA). A repeated measure ANOVA was also performed in order to assess the effect of ROI width and ventricular segment on midwall segmental strain.

## Results

Of the 39 fetuses included in the study, eight examinations (21%) were excluded from the study due to suboptimal visualization of the left ventricular endocardium, leading to unacceptable tracking, yielding a feasibility rate of 79%.

The average frame rate was 109 ± 29 frames per second and the average heart rate 145 ± 29 beats per minute, leading to a frame rate to heart rate ratio of 0.75 frames/s/bpm.

Table 1 shows the change of four chamber longitudinal strain (4ChLS) in accordance to the two different widths and three layers examined. Overall, when changing the ROI width from narrow to wide, 4ChLS decreased from − 20.7 ± 3.6% to − 18.0 ± 4.4% (p < 0.001), respectively. Our results revealed that both ROI width and wall layer significantly impacted strain values, with increasing values when applying narrower ROI and moving from the epicardial to endocardial layer (p < 0.001). There was also significant interaction between the effect of ROI width and wall layer (p = 0.004).

**Table 1:** Layer specific longitudinal strain for narrow, wider and narrow* region of interest (ROI)

Layer	4ch average strain (%) ± SD		
	ROI narrow	ROI wide	ROI narrow*
Endocardial	− 24.5 ± 4.0	− 22.7 ± 4.7	− 24.3 ± 4.3
Midwall	− 20.7 ± 3.6	− 18.0 ± 4.4	− 20.3 ± 4.1
Epicardial	− 17.8 ± 3.7	− 14.8 ± 4.3	− 17.4 ± 4.3
Main p-values from ANOVA p ROI width < 0.001, p Layer specific strain < 0.001, p Interaction between Narrow and Narrow* = 0.004			

*4ch* four-chamber, *SD *standard deviation

Table 2 shows that segmental longitudinal strain also followed the same trend as 4ChLS with falling values as ROI is increased. Our data showed that this was true for all segments (p < 0.001).

**Table 2:** Segmental strain values

Segment	ROI narrow ± SD	ROI wide ± SD	p (α < 0.005) Narrow vs. Wide
Basoseptal	− 16.4 ± 3.0	− 14.7 ± 3.3	< 0.001
Midseptal	− 18.8 ± 3.3	− 16.7 ± 3.7	< 0.001
Apicoseptal	− 23.1 ± 6.7	− 19.8 ± 6.2	< 0.001
Apicolateral	− 23.8 ± 7.1	− 20.1 ± 6.9	< 0.001
Midlateral	− 21.6 ± 5.7	− 18.4 ± 6.5	< 0.001
Basolateral	− 20.1 ± 5.7	− 17.3 ± 6.7	< 0.001

*ROI* region of interest, *SD* standard deviation

We also found increased strain between the segments when moving more apical, as also described by Khan et al. [12] The gradient in strain was valid for both ROI widths (Table 2).

There was no violation of normal distribution and no significant outliers. Both the ROI width and wall layers affected the strain values (Table 1). Overall, for the two widths, strain decreased moving outwards from the endocardial to the midwall layer by 4.2%, with further decrease by 3.0% when moving from the midwall to the epicardial layer (p < 0.001). With regards to the effect of ROI width, changing ROI from the narrow to the wider setting, average strain decreased by 2.5% (p < 0.001). The difference between the strain measures with the initial narrow and the narrow* ROI (obtained by readjusting from wide to narrow) was small (0.3%) and not statistically significant (p = 0.695). Although there was significant interaction (p = 0.004), post-hoc analysis revealed that the trend of falling strain with increasing ROI width held true for all three wall layers, and the trend of falling strain as one moves from the endocardial toward the epicardial layer held true for the examined ROI settings.

The magnitude of the change due to altering ROI width varied depending on layer assessed, with increasing magnitude of change when moving from the endocardial to the epicardial layer. At the endocardial layer, strain decreased by 1.8%, corresponding to a 7.3% proportional change. At the epicardial layer, the corresponding decrease in strain was 3.0%, corresponding to proportional change of 16.9%.

**Fig. 2:**
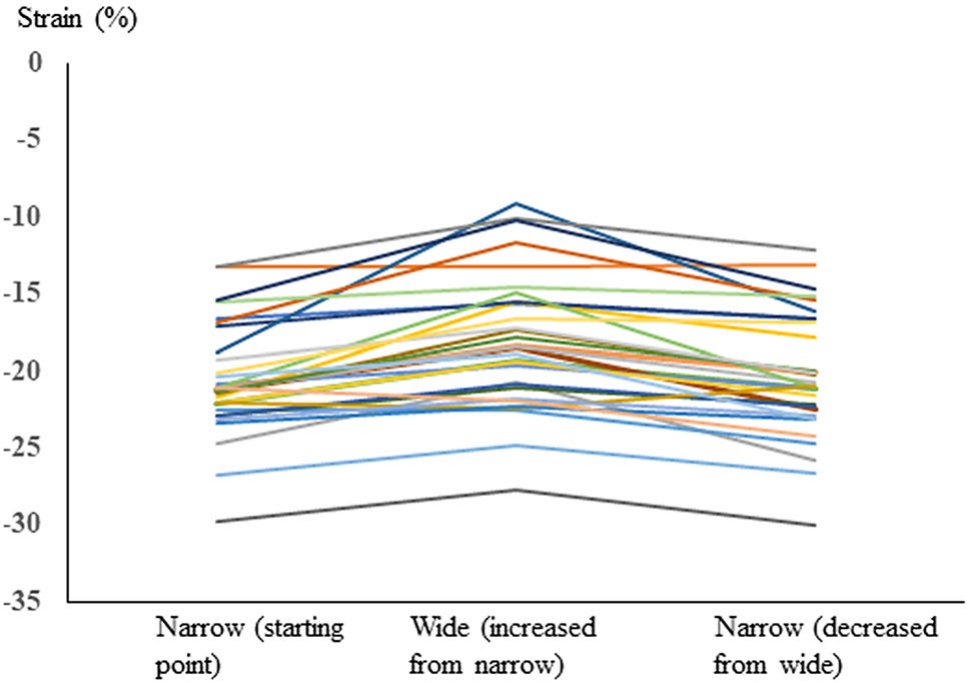
Change in four-chamber longitudinal strain in the 31 fetuses included in the study when changing the ROI width between the widths narrow, wider, and narrow*

The individual changes in strain between narrow, wider and narrow* ROI are shown in Fig. 2.

According to the definitions of Cicchetti et al. [26] both interobserver and intraobserver reproducibility, i.e. reliability, was excellent (Table 3).

**Table 3:** Intra- and inter-observer variability

Layer	Intra-observer	Inter-observer
Endocardial	0.979 (95% CI 0.894–0.993)	0.927 (95% CI 0.820–0.971)
Midwall	0.982 (95% CI 0.924–0.994)	0.951 (95% CI 0.877–0.980)
Epicardial	0.974 (95% CI 0.931–0.990)	0.958 (95% CI 0.897–0.983)

*CI* confidence interval

## Discussion

The main finding of this study was that the definition of ROI width significantly influence measurements of 4ChLS in the fetal heart late in pregnancy. The reduction in strain by increasing ROI width was consistent through midwall, multilayer and segmental analyses (Tables 1 and 2).

Our findings are similar to previous studies in adults. Spriestersbach et al. [18] examined 20 healthy subjects, and Stoebe et al. [19] examined 30 healthy adults and 15 patients with left ventricular dysfunction at different ROI widths, both found strain to decrease with increasing width. When comparing manually adjusted ROI width with default semi-automatic software selected ROI, Otterstad et al. [20] recently reported that increasing ROI width lead to lower strain values.

A recently published review by van Oostrum et al. [8] included 23 studies on STE in fetal hearts. The studies were contradictory concerning development of strain throughout gestation. This was explained by different study design and inconsistent results from varying tracking software and different ultrasound devices. However, another possible explanation for the inconsistencies may be different image acquisition and processing settings. In the present study we show that different ROI widths significantly influences 4ChLS, independent of layer and segment assessed.

At 8 weeks of gestation the structure of the fetal heart is comparable to that of the adult. The contractile fibres of the myocardium are oriented in a helical pattern, in the subendocardium in a right handed helix and in the subepicardium in a left handed helix, whereas the fibres in the midmyocardium are aligned circumferentially [27]. In a complex interplay the myocardial contraction result in a radial thickening and longitudinal shortening with higher strain in the subendocardium compared to the mid- and subepicardium [28], as well as increased strain moving from the basis to the apex [13]. In this study we confirm this also in the fetal heart. With increased ROI-width we show decreased strain both between layers, segments and in 4ChLS which is explained through involvement of myocardial fibres with less deformation.

Echocardiography during fetal life is challenging compared to the post-natal period. The thickness of the maternal abdominal wall and/or unfavourable fetal position or movements may hamper ultrasound examination of the fetal heart. As the beam must traverse through both maternal and fetal tissue to reach the fetal heart, low-frequency probes must be used, which result in reduced spatial resolution. In addition, one must bear in mind that the fetal myocardium is quite thin (width ranging from 2.4 to 4.2 mm at 37 weeks gestation) [29]. The abovementioned factors pose challenges in fetal echocardiography and in speckle tracking echocardiography in particular [30].

In this study, we assessed multi-layer strain. Previous studies have shown its feasibility in older patients in the case of myocardial toxicity [31], aortic stenosis [32], and ischemic cardiomyopathy [33, 34]. However, the accuracy of multi-layer strain measurements remains disputed [35].

Although we see that ROI width affects strain values, it is not possible to determine which ROI settings yield the most accurate strain values as this would require a reference method to serve as a source of comparison. Although previous studies have examined the effect of ROI width as mentioned above [18−20], there is a shortage of studies that use a reference method. Cardiac MRI could have been an option, but the use in fetuses is still hampered by challenges in spatial and temporal resolution [36].

The ROI widths narrow and narrow* resulted in different numerical strain values in spite of ROI-widths being the same. Although this difference was not statistically significant (p = 0.425), nor of clinical relevance, it is nonetheless an interesting finding as it highlights that strain not only depends on the ROI-width, but also the initial ROI-width.

The most important strengths of the study were that analyses were carried out by one experienced cardiologist, and with excellent intraobserver variability, and that all the ultrasonographic examinations were performed at the same gestational age (37 weeks of gestation).

There are limitations to this study. We evaluated strain using equipment and software from a single vendor. Inter-vendor discrepancy in strain remains an on-going challenge for STE [8], although efforts are being made at achieving some form of standardization across vendors [37]. Strain was assessed in the four-chamber view only, not from three apical views, as recommended for adults in the joint consensus paper by the European Association of Cardiovascular Imaging and the American Society of Echocardiography [24]. As previously noted, echocardiographic evaluation of the fetal heart remains challenging, particularly for the 2-chamber and 3-chamber views, due to loss of the endocardial borders of the septal and lateral walls [38]. Another limitation is the fact that only fetuses at week 37 of gestation were examined. At this age, one could argue that rather than acquire advanced measures of ventricular function one could simply deliver the fetus and treat the neonate. However, we hope our study will highlight an important aspect of fetal imaging.

In conclusion, this is to the best of our knowledge the first study to examine the effect of different widths of the ROI when assessing fetal strain late in pregnancy. Strain assessment is feasible and highly reproducible in this age group. We found that the setting of ROI width significantly affected STE derived left ventricular longitudinal strain values in fetuses. Although the effect was greatest in the epicardial layer, a reduction in strain was consistent through midwall, multilayer, and segmental strain across the entire myocardium. Fetal cardiologists and obstetricians should be aware of the effect of different ROI widths as a source of variation in strain measurements when assessing strain. A standardization of the ROI setting would strengthen the method as a routine measure of ventricular function in the fetus.

## Data Availability

Data available on reasonable request from authors.
